# Root-Crown Ratio in Permanent Dentition Using Panoramic Radiography in a Selected Iranian Population

**Published:** 2014-12

**Authors:** Sina Haghanifar, Ehsan Moudi, Shirin Abbasi, Ali Bijani, Arash Poorsattar Bejeh Mir, Nafiseh Ghasemi

**Affiliations:** a Dept. of Oral and Maxillofacial Radiology, School of Dentistry, Babol University of Medical Sciences, Babol, Iran.; b Dept. of Oral and Maxillofacial Radiology, School of Dentistry, Babol University of Medical Sciences, Babol, Iran.; c General Dental Practitioner, Private Practice, Babol, Iran.; d Noncommunicable Pediatric Diseases Research Center, Babol University of Medical Sciences, Babol, Iran.; e Dental Materials Research Center, School of Dentistry, Babol University of Medical Sciences, Babol, Iran.

**Keywords:** Panoramic radiography, Root-Crown Ratio, Anatomy

## Abstract

**Statement of the Problem:** Determining the crown-root ratio is crucial in many dental clinical decisions. There are no reliable data presented for Iranian population.

**Purpose: **The aim of this study was to assess the root-crown ratio of permanent teeth with regards to the relationship of gender and jaw type using panoramic radiographs. The reliability of method was also assessed.

**Materials and Method:** The crown and root lengths of teeth were measured by a digital caliper on panoramic radiographs of 185 individuals except for the third molars. A total of 1994 teeth was studied and 50.8% of the teeth belonged to males and 49.2% belonged to females. The modified Lind method was applied.

**Results:** The highest mean root-crown ratios in both arches of both genders were obtained in the second premolars, followed by the first premolars and canines. In both genders, the lowest root-crown ratios were found in the maxillary central incisors. In male patients, the mean root-crown ratio was higher (p= 0.003) than that of females. Using Bland-Altman analysis, a non-significant difference of 0.006 (95% CI: -0.012-0.024) and 0.0002 (95% CI: -0.011-0.011) were found for intra-observer and inter-observer agreement, respectively.

**Conclusion:** Assessment of the root-crown ratio in permanent dentition could be performed on panoramic radiographs with acceptable reproducibility in an Iranian Population. Considering observed differences, our findings suggest that for the accurate assessment, this ratio must be calculated for male and female patients and also for the upper and lower jaws, separately.

## Introduction

In radiographic studies, measuring the root-crown (R/C) ratio is more beneficial than assessment of the absolute linear R/C measurement since a change in radiographic angulation affects the radiographic tooth length, while its effect on R/C ratio is negligible. In fact, this ratio remains stable when antero-posterior or latero-lateral cants in certain ranges occur.[1] Horizontal and vertical distortion may still happen even if the dentition is well situated within the focal trough at the time of exposure. To overcome the vertical distortion in the radiographic assessment, linear calculation might be advantageous. The magnification of panoramic radiographs taken with different machines and also the magnification between different regions of the same radiograph may vary. Since root and crown are usually in the same vertical plane, the magnification factor cannot exert any major effect on the R/C ratio.[2]

Short-root teeth with low R/C ratios are effective in the prognosis and treatment planning of many dental procedures.[1] The etiology of developmental short-root teeth can be attributed to genetic or extrinsic factors such as chemoradiotherapy, syndromes, or systemic diseases.[1] The abnormal short root usually occurs in the maxillary central incisors with an incidence rate of 1–10%.[3]

Some studies have previously performed to investigate the clinical usefulness of the panoramic views to assess R/C ratios. Stramotas et al. performed a study in 2000 to assess the reliability of crown height, root length, and R/C ratio. Moreover, they evaluated the angulations of teeth relative to each other and hypothetical reference lines in the same segment on panoramic radiographs. They reported that tooth lengths and R/C ratios were reliable and could be measured accurately.[2]

Holta et al. showed that the assessment of the R/C ratio in panoramic radiography could be performed with an acceptable reproducibility and panoramic radiographs could be used to determine the progression of apical root resorption in orthodontic patients and other cases.[1]

In real practice, it is not possible to measure the crown and root lengths directly as the tooth is embedded in the alveolar socket. Hence, clinician’s decisions are mainly based on indirect estimation of lengths and ratios. Among current available radiographic modalities, panoramic radiography is used extensively. Considering the imperative role of radiography especially panoramic radiography in dentistry, and also the significance of assessing root length and apical root resorption in orthodontic and prosthodontic patients, the present study was performed to survey the R/C ratio and to assess the reproducibility of findings in a selected Iranian population. 

## Materials and Method

In this cross-sectional study, non-random sampling was performed on panoramic radiographs of patients with permanent dentition, collected from two private oral and maxillofacial radiology clinics during spring and summer of 2009. On each radiograph, teeth with extensive dental caries or restorations, root resorption, incomplete root development, severe crowding, considerable attrition, intrabony lesions attached to root, hypercementosis and dilacerations were excluded. In addition, the third molars were not taken into account. Radiographs of the patients with a medical history of head and neck irradiation, chemotherapy, genetic disorders related to developmental tooth anomalies (such as Down and Turner syndromes), trauma, cleft lip, and cleft palate were not assessed. 

**Figure 1:**
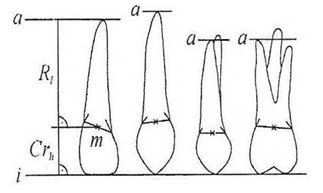
Modified Lind method. i:incisal (occlusal in premolar and molar teeth) level, a: apical level, Rt: root height, Cr_h_: crown height, M: midpoint of a straight line that connects the points of intersection between the outer contours of root and crown

In this study, 1994 teeth were assessed separately by two blinded maxillofacial radiologists (A and B, inter-observer assessment). After three months, a repeated measurement was performed by observer A on 10% of radiographs that were selected randomly (A` intra-observer assessment). Since the cemento-enamel junction (CEJ) level is not clearly visible on panoramic radiographs for measuring the crown height and root length of each tooth, modified Lind technique was adopted to determine the midpoint of crown and root, therefore, the length of each tooth could be measured accurately.[ 4 ] Based on this technique, the midpoint of a straight line that connects the points of intersection between the outer contours of root and crown was selected as point M (Figure 1).

In addition, an occlusal reference line for incisors or molars was placed to follow the incisal edge or to connect the buccal cusps. Furthermore, in canines or premolars, a line that formed a tangent to an incisal tip or to a buccal cusp was placed perpendicular to the long axis of the tooth. In order to determine the apical reference line in three-rooted teeth, after the exclusion of the palatal root, the longest buccal root was selected, while in two-rooted teeth, the longest root was selected. Palatal roots were excluded due to its unsatisfactory resolution in panoramic radiography which was explained formerly by Lind.[ 4 ] Then, a line tangent to the apex of the longest root and parallel to incisal/occlusal reference line was placed and considered as the apical reference line. The length of the perpendicular line from point M to the incisal/occlusal reference line was considered to be equal to the root length. The crown height was equal to the length of the perpendicular line from point M to the apical reference line. The difference between the points and reference lines was measured by a digital caliper (Goanjigo SR 44, China) with an accuracy of ±0.01 mm. The measurements were performed on 185 (out of total 350) panoramic radiographs and 1994 teeth of male (50.8%) and female (49.2%) patients.

Numeric data were expressed as means (standard deviations or standard errors). Nominal data were displayed with frequencies (%). Comparisons of mean R/C ratios between genders and jaws were accomplished with t-test. 

To test the method error (uncertainty associated with observers errors), 308 radiographs were re-checked. Inter-observer and intra-observer agreements were calculated by plotting mean measurements against corresponding differences (Bland-Altman nomogram). A further Pitman’s test of differences was also applied to test the agreements. A two-tailed *p*< 0.05 was considered statistically significant. SPSS 19 and STATA 11 software programs were used.

## Results

The highest mean R/C ratios in both arches of both genders were obtained in the second premolars, followed by the first premolars and canines. In both genders, the lowest R/C ratio was found in the maxillary central incisors. In females, the lowest R/C ratios were recorded in the maxillary first molars and the maxillary lateral incisors, while in males, the lowest ratios were found in the maxillary lateral incisors followed by the mandibular central incisors. Descriptive statistics (means, standard deviations and 95% confidential intervals) are presented in Table 1.

**Table 1:** Mean root- crown ratio in males and females separated for each jaw for permanent dentition with standard deviation (SD) and 95% confidence intervals (CI)

**Jaw**	**Tooth**	**Gender**	**No.**	**Mean**	**Standard** **Deviation (SD)**	**Confidence** **Interval (95% CI)**	P value*	**Standard** **Error (SE)**	P value**
Maxilla	Central	Female	78	1.71	0.36	1.63-1.79	0.03	0.19	0.94
		Male	79	1.71	0.31	1.63-1.78	0.001	0.19	
	Lateral	Female	75	1.77	0.28	1.70-1.83	0.02	0.2	0.55
		Male	67	1.80	0.33	1.72-1.88	0.03	0.2	
	Canine	Female	57	2.20	0.31	2.12-2.29	0.009	0.29	0.18
		Male	70	2.28	0.32	2.20-2.36	0.13	0.27	
	First Premolar	Female	48	2.30	0.40	2.19–2.42	0.01	0.33	0.93
		Male	58	2.31	0.37	2.21-2.41	0.003	0.30	
	Second Premolar	Female	62	2.53	0.37	2.43–2.62	0.61	0.32	0.72
		Male	60	2.55	0.22	2.49–2.61	0.40	0.21	
	First molar	Female	63	1.73	0.23	1.67–1.79	0.001	0.25	0.001
		Male	63	2.01	0.31	1.94–2.09	0.001	0.24	
	Second molar	Female	72	2.08	0.32	2.00–2.15	0.40	0.24	0.16
		Male	74	2.15	0.34	2.07–2.23	0.24	0.2	
Mandible	Central	Female	80	1.85	0.42	1.75–1.94		0.2	0.86
		Male	82	1.86	0.39	1.77–1.95		0.19	
	Lateral	Female	95	1.88	0.34	1.81–1.95		0.19	0.40
		Male	95	1.93	0.42	1.84–2.02		0.3	
	Canine	Female	61	2.36	0.30	2.28–2.43		0.3	0.8
		Male	62	2.37	0.33	2.28–2.46		0.3	
	First Premolar	Female	84	2.49	0.37	2.40–2.57		0.27	0.85
		Male	80	2.50	0.34	2.42–2.57		0.27	
	Second Premolar	Female	71	2.56	0.33	2.48–2.64		0.3	0.24
		Male	73	2.59	0.38	2.51–2.68		0.3	
	First molar	Female	66	2.23	0.34	2.14–2.31		0.27	0.99
		Male	68	2.23	0.39	2.13–2.33		0.27	
	Second molar	Female	70	2.03	0.27	1.97–2.10		0.24	0.001
		Male	81	2.22	0.32	2.15–2.29		0.24	

* Comparison of antagonist teeth, upper jaw against lower jaw.

** Comparison of similar teeth between males and females.

Mandibular teeth, except for second molars and second premolars in both genders and canines in males, showed substantially higher R/C ratios compared to their antagonistic maxillary teeth. In males, the mean R/C ratios in maxillary first molars and mandibular second molars were considerably higher than those in females. The mean R/C ratio in males [2.11 (0.44), 95% CI: 2.08-2.13] was significantly higher than that in females [2.17 (0.44), 95%CI: 2.14-2.19] (*p*= 0.003).

**Figure 2:**
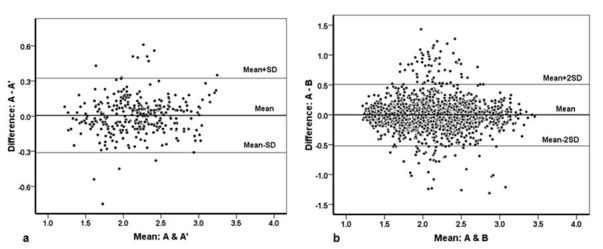
a:  Bland-Altman plot displaying intra-observer reliability for observer A and the repeated test (A`).  b: Bland-Altman plot displaying inter-observer reliability for observers A and B

Using Bland-Altman analysis, a non-significant difference of 0.006 (95% CI: -0.012-0.024) and respective upper and lower bands of -0.312 and 0.324 were found for repeated measures of observer A (A, A`). Pitman’s test revealed a high agreement level (r=0.107, *p*= 0.62). In addition, a non-significant difference was found for inter-observer agreement (A, B) with a mean difference of 0.0002 (95% CI:-0.011-0.011) and respective upper and lower bands of -0.505 and 0.506. Moreover, Pitman’s test showed insignificant differences (r=-0.037, *p*= 0.99). Intra- and inter-observer reliability is shown in Figures 2.

## Discussion

In this study, tooth lengths and the R/C ratios were obtained using panoramic radiography and the method reproducibility was assessed. It was shown that the R/C ratio was higher in males than in females and the mandibular teeth were longer than maxillary teeth. This method exhibited considerable reliability in terms of both inter- and intra-observer reliability.

Panoramic radiography is of great importance in the treatment planning stage and during the patient follow-up because it is reproducible and causes less patient exposure and most importantly, in prosthodontic and orthodontic studies despite the magnification differences for separate measures of root and crown lengths in anterior and posterior segment of radiographs, the R/C ratio remains the same. The R/C ratio is reduced following a decrease in the root length in the instances such as bone marrow transplant, chemotherapy, cleft palate, and trauma cases.[2, 5-6] Trauma with crown fracture, attrition, and dental caries results in an increase in the ratio. Al-Jamal et al. reported that the effect of cleft palate on adjacent teeth, particularly incisors and canines, and incomplete root development may cause an increase in the R/C ratio.[5] However, developmental root anomalies such as defect in size and the number and morphology of teeth have an impact on all the teeth. In hypodontia, for instance, bilateral developmental asymmetry has been reported without presence of any syndrome or known etiologic factor (such as cleft palate).[7]

The R/C ratios, obtained by panoramic radiography, are applied routinely in the evaluation of root resorption in orthodontic treatments for estimation of root support in anchorage cases, the treatment planning, and the evaluation of prognosis of prosthodontic treatments.[1] Rowland et al. assessed the R/C ratio by Kunosand Simpson method and reported that it was possible to predict eruption time of mandibular second premolars.[8] Crown morphologic features could predict the root morphology with high accuracy (approximately 95 percent).[9] In addition, root-crown ratio is a significant survival predictor especially when multidisciplinary prosthodontic, periodontal, and endodontic treatments are planned.[10]

There is some dissimilarity for the highest and lowest R/C ratios reported in the literature. In the present study, the highest R/C ratios were recorded in the second premolars, the first premolars and the canines. Similarly, Holta et al. obtained the same results in Finland.[1] Midtbo et al. reported the highest R/C ratio for the mandibular first and second premolars.[11] The lowest R/C ratio in both genders was recorded in the maxillary central incisors (1.71), followed by the maxillary first molars (1.73) and the maxillary lateral incisors (1.77) in females. Midtbo et al. reported the lowest R/C ratio in the maxillary central incisors (1.68).[11] Meanwhile, Holta et al. reported the least R/C ratio in the maxillary central incisors in females (1.78), followed by the maxillary first molars in females (1.80) and the maxillary central incisors in males (1.86).[1] Although the lowest R/C ratios in the present study and Holta et al.'s study were found in the same teeth, these ratios were higher in Holta et al.'s study.

The R/C ratio is highly dependent on crown length and/or root length so that even a one-millimeter error in the determination of the accurate inter-section point of crown and root could cause a significant difference in this ratio. Thanyakar et al. and Larheim and Eggen reported that the difficulty in the determination of reference points was the main reason for errors in vertical measurements on panoramic radiograph. They also concluded that the inter- and intra-observer differences had to be taken into consideration when analyzing the radiographic effects of orthodontic treatment.[12-13]

In the present study, the mean R/C ratios in males and females were 2.17 and 2.11, respectively, which are close to the results achieved by Holta et al. who reported to be 2.10 and 2.07 in male and female patients, respectively. 

In the present study, the mean R/C ratios of all the teeth in males (except for maxillary central incisors and mandibular first molars) were higher than those in females and this difference was highly significant in maxillary first molars and mandibular second molars. Lind and Jackobson et al. did not report any significant differences between the two genders in the R/C ratios of maxillary central incisors.[4, 14] Conversely, Holta et al. reported a significant difference between the two genders in the R/C ratios for maxillary incisors, laterals, and molars in addition to mandibular incisors.[1] In this study, the difference in the mean R/C ratios in all the teeth between both genders was statistically insignificant, which might be attributed to the lower number of samples in this study compared to that of Holta et al. study (2779 teeth). Hence, with an increase in the number of samples, small changes might have resulted in significant differences in the results. In the present study and the study carried out by Holta et al., the mean R/C ratio in males was significantly higher than that in females. Considering the significant similarities between the results reported in this study and in the literature, it might be concluded that the R/C ratio does not vary significantly among different races.

The present study showed that the mean R/C ratio of all the mandibular teeth, except for the second molars in females, was higher than that of maxilla. This difference was significant, except for the second premolars in both genders and canines and the second molars in males. This was in agreement with the study by Holta et al. who showed that the mean R/C ratio of all the mandibular teeth, except for lateral incisors in males and the second molars in both genders, was also substantially higher than that in maxillary teeth.[1]

The findings of the present and Holta et al.'s studies, who determined the tooth length based on tooth morphology (outer contour), were more similar than the findings reported by Bjorndal et al., Carlson et al., Midtbo et al., and Jackobson et al. who used CEJ to determine the midpoint of crown and root. In the assessment of extracted teeth, when CEJ is used to determine the midpoint of crown and root, the crown is larger and the R/C ratio is smaller than those of the present study.[1, 11, 14-16]

We experienced that the standard error of the R/C ratios gradually increased from the anterior teeth to premolars and then it decreased in such a way that the smallest statistical dispersion, which was indicated by the standard error, corresponded to central and lateral teeth with the highest statistical dispersion to canines and premolars. These results are highly consistent with those reported by Holta et al., who reported the highest continuous error values. Holta et al. analyzed the value of the intra-observer error for each tooth and compared it with the biological deviation.[1] The maximum error value in the premolar region is attributed to the presence of maxillary sinus and the overlap of premolars in the mandible. Therefore, in the present study, the above-mentioned maximum error value might be mainly due to the error in observer measurements and not to the variability and biological errors. However, more research and studies in this field are required.

It is noteworthy to mention that the measurement of crown-root or root-crown ratios has also some disadvantages. For instance, in microdontia, while the same R/C ratio is maintained, the crown and root are smaller than their normal sizes and the correct R/C ratio does not reflect the suitable size and diameter of root for sufficient tooth support.[1]

The present study revealed that panoramic radiography was reproducible and reliable in determining root length. The intra-observer reproducibility and the inter-observer reproducibility, as shown in Figure 1 and 2, were neither scale nor observer dependent and no substantial outliers were presented. These reassure the reliability of the method. The results are consistent with the findings of Holta et al. who employed the same method.[1]

Concerning the limitations of this technique, the unequal magnification in the anterior and posterior areas of panoramic radiographs has always been challenging for researchers and clinicians in assessing tooth lengths and positions. The extensive horizontal dimensional change makes the panoramic radiography unreliable, however, the vertical dimensional change is considerably smaller than the horizontal one and is reliable if the patient’s head is placed correctly and the tooth is in the focal trough. 

Although the total tooth length is affected by vertical dimensional change, sustained C/R ratio in the anterior and posterior regions has extended its clinical application. While maintaining total length of tooth, changes in the occlusal plane (particularly in the mesio-distal plane) adversely affect the C/R ratio. These changes are less significant in the occlusal right and left head tilt of a patient and the projections with extensive antero-posterior rotation and right and left displacements were not assessed in the current study.[17-18]

 Moreover, the tooth inclinations and their effects on C/R ratios were not assessed in this study. This ratio is changed subsequent to an alteration in the bucco-lingual inclination distance from focal trough, tooth length, and vertical depth of impaction. 

## Conclusion

The measurement of R/C ratio in permanent teeth on panoramic radiographs could be performed with good reproducibility. Therefore, evaluation of the amount of root resorption on panoramic radiographs during treatment is possible. Except for the second premolars and the second molars in both genders and canines in males, the mean R/C ratio of mandibular permanent teeth was higher than those in the maxilla in both genders. The mean R/C ratio of the maxillary first molars and the mandibular second molars and the total mean R/C ratio were significantly higher in males than those in females. The results suggest that the gender of patient and the tooth type (the type of jaw) should be taken into account while assessing the R/C ratios. 
